# GPT-4’s capabilities for formative and summative assessments in Norwegian medicine exams—an intrinsic case study in the early phase of intervention

**DOI:** 10.3389/fmed.2025.1441747

**Published:** 2025-04-10

**Authors:** Rune Johan Krumsvik

**Affiliations:** Department of Education, University of Bergen, Bergen, Norway

**Keywords:** GPT-4, formative assessment, summative assessment, final exams, medicine, case study, educational innovation, patient cases

## Abstract

The growing integration of artificial intelligence (AI) in education has paved the way for innovative assessment methods. This study explores the capabilities of GPT-4, which is a large language model (LLM), on a medicine exam and for formative and summative assessments in Norwegian educational settings. This research builds on our previous work to explore how AI, specifically GPT-4, can enhance assessment practices by evaluating its performance on a full-scale medical multiple-choice exam. Prior studies have revealed that LLM’s can have certain potential in medical education but have not specifically examined how GPT-4 can enhance formative and summative assessments in medical education. Therefore, my study contributes to filling gaps in the current knowledge by examining GPT-4’s capabilities for formative and summative assessment in medical education in Norway. For this purpose, a case study design was employed, and the primary data sources were 110 exam questions, 10 blinded exam questions, and 2 patient cases within medicine. The findings from this intrinsic case study revealed that GPT-4 performed well on the summative assessment, with a robust handling of the Norwegian medical language. Further, GPT-4 demonstrated a reliable evaluation of comprehensive student exams, such as patient cases, and, thus, aligned closely with human assessments. The findings suggest that GPT-4 can improve formative assessment by providing timely, personalized feedback to support student learning. This study highlights the importance of both an empirical and theoretical understanding of the gap between traditional assessment methods and educational practices and AI-enhanced approaches—particularly the importance of the ability of chain-of-thought prompting, how AI can scaffold tutoring, and assessment practices. However, continuous refinement and human oversight remain crucial to ensure the effective and responsible integration of LLM’s like GPT-4 into educational settings.

## 1 Introduction

The increasing incorporation of artificial intelligence (AI) in education has opened up new avenues for innovative assessment methods and educational practices. Effective assessment is crucial for evaluating student learning processes, ensuring academic rigor, and enhancing educational outcomes. Several meta analyses reveal that Large Language Models (LLMs) can enhance feedback practices and academic performance (1, 2). The potential of AI, particularly GPT-4, to transform assessment practices is underexplored—particularly in Norway—and requires further research, including its impact on the Norwegian language.

Assessments that utilize AI can significantly shape the quality of education and the learning experiences of students. The interaction between AI-driven assessments and traditional methods is a key area of interest, particularly as educational institutions seek to incorporate advanced technologies such as adaptive learning tools, large language models, etc. into their practices. Internationally, we find that there is an increasing research interest in this topic and, thus, a growing number of research studies (1–3). Building on these studies and our previous research on AI in education (4–8), this case study aims to examine GPT-4’s capabilities for formative and summative assessments in Norwegian educational settings. The research focuses on addressing the following questions:

1. *What is the capability of GPT-4 on a medicine exam in Norway?*
2. *How can GPT-4’s capability be applied in formative and summative assessments for students?*

By investigating these questions, the case study seeks to understand the potential of GPT-4 to transform assessment practices—thereby bridging the gap between traditional methods and AI-enhanced approaches—and identifies areas for further innovation in educational assessment.

### 1.1 Background

This article began as part of a book project in 2022 (6) and coincides with the launches of ChatGPT in November 2022 and GPT-4 in March 2023. The article examines the impact of the technological shift as observed in 2023 and early 2024, focusing on the differences between the *real* and *perceived* affordances (28) of AI—particularly language models—in education and healthcare. Further, it highlights the opportunities, challenges, dilemmas, and risks of this transition. In this context, I explore GPT-4’s capabilities in medical education based on the current state of knowledge. However, the current state of knowledge regarding the use of AI in medical education remains limited, with much of the existing literature dominated by editorials, pre-prints, and small-scale studies. While these contributions have offered valuable initial insights, there remains a lack of comprehensive, large-scale research on how LLM’s, such as GPT-4, can be integrated effectively into medical educational contexts (6). Given this gap, this case study is exploratory and addresses a completely new research focus within the Norwegian educational context (and in Norwegian), the current knowledge base is still very limited in relation to the scope of this article. Therefore, this article focuses strictly on the relevant research within its defined scope, without drawing on broader knowledge areas (such as general benefits of AI, medical benefits of AI, etc.). This focused approach is essential to ensure that the findings directly contribute to the specific topic of GPT-4’s capabilities for formative- and summative assessment in the context of the study, where the knowledge base is currently underdeveloped. As such, the brief knowledge summary below reviews research with relevance for this explorative case study.

### 1.2 Summary of knowledge

Gilson et al. (10) demonstrated ChatGPT’s capability in medical question-answering tasks, achieving accuracy comparable to a third-year medical student on the NBME-Free-Step-1 dataset. Kung et al. (11) further validated these capabilities by showing ChatGPT scoring near the passing threshold across all steps of the United States Medical Licensing Exam (USMLE), highlighting the model’s potential to support medical education through insightful explanations.

Artsi et al.’s (12) systematic review confirmed that Large Language Models (LLMs), including GPT-4, are capable of generating valid medical exam questions, especially multiple-choice questions (MCQs). However, the review emphasized that questions generated by LLMs often required further revision, underscoring the models’ limitations and the necessity for supplementary human oversight.

Grévisse (13) provided evidence that GPT-4 can reliably grade student answers in medical education but noted the importance of human oversight due to potential grading biases. Madrid et al. (14) further supported these findings, demonstrating GPT-4’s successful integration with external plugins, enhancing its performance on medical board examinations and indicating ongoing model improvement by developers. And a review by Ray (3) demonstrated GPT models’ proficiency on the United States Medical Licensing Exam (USMLE).

While these studies collectively highlight the promising capabilities of GPT-4 and related models, they have not specifically addressed GPT-4’s potential to enhance formative and summative assessments in medical education within the Norwegian context. Therefore, this study aims to fill this specific knowledge gap by examining GPT-4’s capabilities for formative and summative assessments in Norwegian medical education, providing insights that can directly inform future practices.

### 1.3 Theoretical framework

As outlined by VanLehn (15), Intelligent Tutoring Systems (ITS) have historically played a crucial role in providing personalized learning experiences, functioning as mediating tools between students and instructional content in specific subject disciplines. The backdrop for this case study raises several intriguing questions regarding the evolving role of intelligent tutoring systems (ITS) in modern education. In the age of AI, these systems may evolve significantly, opening up new possibilities driven by recent advancements, particularly the development of sophisticated models like GPT-4. With its advanced natural language processing capabilities, GPT-4 has the potential to redefine the role of ITS across various subject disciplines. By leveraging this technology, ITS has the potential to significantly enhance both formative and summative assessments, enabling possibilities previously unattainable—a recent meta-analysis of ITS further supports this, showing promising results (16).

These possibilities suggest that the fusion of ITS with AI might open new avenues for personalized education, but they also prompt us to consider the broader implications for assessment practices and student learning outcomes. Formative assessments, designed to enhance ongoing student learning through regular feedback, open up new possibilities for students to receive real-time contextually relevant feedback when integrated with GPT-4’s ability. Could GPT-4 offer more adaptive and personalized pathways, making formative assessments not merely tools for evaluation but those for continuous improvement? Similarly, summative assessments— which focus on measuring cumulative knowledge at the conclusion of an instructional period—may be transformed through GPT-4’s capacity to function as an interactive “sparring partner.” How might this dynamic interaction with previous exam sets reshape student preparation for final evaluations? Could it foster deeper engagement and feedback by mimicking real exam scenarios and scaffold students based on their performance? This case study aims to provide a few insights into these important questions using the concept of feedback as an underlying “lens”.

Feedback has been identified as a highly influential factor in improving student learning outcomes, with a substantial effect size, as demonstrated by numerous meta-analyses (17, 18). This highlights the potential of feedback to significantly enhance student learning. Thus, the focus here is on assessing GPT-4’s ability to provide summative feedback on a medicine exam (summative assessment) and exploring how its capabilities can be applied in formative assessments. Drawing on Hattie and Timperley’s (17) educational feedback theories—particularly their concepts of *feed up*, *feedback*, and *feed forward*, and the coherence among these elements—provides valuable “lenses” for bridging the theoretical foundations with GPT-4’s capabilities in both summative and formative assessments for medical students.

*Feed-up* involves clarifying learning objectives and expectations for students to ensure that they understand the direction of their learning (often outlined as learning outcomes in course descriptions and similar materials). This helps align their efforts with the desired outcomes and guides their performance and improves learning efficacy. In the context of this study, the feed-up process is attached to the exam questions, providing students with a clear understanding of the goals and standards they need to achieve. Previous exam papers (as used in this case study) are important in this formative assessment process, and these include exam questions that have previously been used in the same medical exam and are available to medical students who wish to practice for upcoming exams. *Feed up* in this case study is thus related to the medicine exam set that consisted of 110 exam questions in Norwegian language (MD4062) from 2020.

*Feedback* is a critical component of formative assessment, providing students with information regarding their current performance relative to the learning goals. Effective feedback and formative assessments help students identify their strengths and areas for improvement, ultimately enhancing their understanding and performance. In this study, GPT-4’s ability to generate feedback on the summative assessment (feed up) attached to the first research question was evaluated using 110 exam questions and 10 blinded exam questions. Sections 3.1 and 3.2 highlight how GPT-4 responds to these questions and offers detailed and relevant feedback as a possible sparring partner in student learning.

*Feed-forward* is a proactive approach that complements traditional feedback by focusing on future learning strategies and improvements. It involves providing formative assessments along with recommendations to guide students in enhancing their future performance. Based on research question 2 and detailed in section 3.3, this study evaluates GPT-4’s ability to handle formative assessments through chain-of-thought prompting. By assessing authentic cases (cases 1 and 2), GPT-4 offers insights and formative assessments that can aid students in understanding how to enhance their learning and performance in subsequent tasks.

In summary, the case study examines if the integration of GPT-4 in medical exams and educational contexts can be theoretically justified by this educational feedback framework.

## 2 Methodology

Since this study examines GPT-4’s capabilities, it is important to highlight what distinguishes GPT-4 from earlier versions, particularly ChatGPT. Both GPT-3 and GPT-4 are general-purpose language models developed by OpenAI, capable of performing a wide range of tasks, such as text generation, translation, and problem-solving. On the other hand, ChatGPT is not a separate or fine-tuned version of GPT-4 but rather a specific application of the GPT-4 model, optimized for conversational interactions. It utilizes the capabilities of GPT-4 to maintain coherence in dialog-based scenarios but is powered by the same underlying model, GPT-4, which can be applied in many other contexts beyond chat. For example, can GPT-4 be used to write essays, generating code, summarizing articles and assist in legal document drafting, where its capacity for language understanding is applied differently from conversational chat. GPT-4 is particularly effective when using chain-of-thought prompting, which is a technique used in AI language models in which a series of intermediate reasoning steps are provided to the model to help it generate more accurate and logical responses. By breaking down a complex problem into smaller, more manageable parts, GPT-4 can better understand the context and produce a coherent solution. This method enhances the model’s ability to handle intricate queries by simulating a step-by-step thought process.

This case study discussed here is intrinsic and exploratory in nature (19, 20) and examined GPT-4’s capabilities in the field of medical education. The 110 exam questions are not collected from any participants, but derived from the university’s official guidance; moreover, it is only GPT-4 that has been providing the answers and explanations for the exam. The 110 exam questions are based on a past exam paper from an identical medicine exam (MD4062) in 2020 (2020 - IIID - MD4062 - Exam 1, held on 2020-05-11; see here). These past exam papers are publicly available to medical students who wish to practice for upcoming exams.

**Figure 1:**
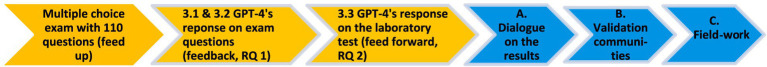
The research process of the intrinsic case study. The yellow arrows represent the main parts of the study with the theoretical concepts included and the three main phases of the study (RQ1, Sections 3.1 and 3.2 as well as RQ 2 and Section 3.3) with the data sources. The blue arrows represent the supplemental data in this article.

I conducted a cumulative data collection and analysis process (21) with the theoretical concepts *feed up*, *feedback* and *feed forward* intertwined (see Figure 1), basing the 110 multiple exam questions (1, feed up) on *chain-of-thought prompting* with the exact same wording as that in the text of Norwegian exam questions. The 110 multiple choice exam questions and 10 questions with blind alternatives (RQ 1, Sections 3.1 and 3.2; feedback) were developed on the basis of prior *chain-of-thought prompting*, and the test results from the national laboratory (RQ 2, Section 3.3; feed forward) were based on earlier *chain-of-thought prompting* (see Appendix 1).

Further, in the supplementary data collection process (blue arrows), I integrated the research questions (RQ 1 and RQ 2) in the dialog (A) of the results from Sections 3.1–3.3 with the interaction with GPT-4. Then, several validation communities (B) (one researcher, one medicine student, one medicine doctor, and one referee in a scientific journal) were applied to check the preliminary finding. Finally, I conducted further field (C) work to check for possible biases and misinterpretations as well as check the finding in light of the abovementioned knowledge summary within this field.

The main test period was from March 25, 2023 to August 5, 2023 and this medicine exam is 30 ECTS and includes a written school exam of 4 hours, with a weightage of 1/3 (of total marks); moreover, I tested the Norwegian language version of this exam (MD4062) from 2020. The MD4062 is a sixth-year medical school exam in which the exam questions are multiple choice and the grading of this written school exam is only in terms of “pass” or “fail.” No printed or handwritten aids are allowed; a specified, simple calculator is permitted.

In addition to this written school exam, the students have two oral exams of 2 hour each, weighting 1/3 each, and no aids are permitted to be used. The oral exams are also graded in terms of “pass” or “fail.” In this case study, only the written school exam has been investigated (for more information regarding this exam, see Course - Final Comprehensive Medical Exam - MD4062 - NTNU).

Data analysis, step 1, was based on the percentage score of the entire test battery. Data analysis, step 2, involved 10 selected case tasks tested with GPT-4, based on a total of 110 exam questions, where no multiple-choice options were provided (blinded alternatives). Data analysis, step 3, involved GPT-4 analysis of two authentic patient cases based on actual laboratory data and evaluated GPT-4’s ability to provide authentic health recommendations based on the two patient cases from a national laboratory^1^ in Norway. The supplemental data was collected from August 2023–March 2024 and consisted of comprehensive interactions with GPT-4, the use of validation communities, and fieldwork (described above).

## 3 Results

The results of the main data sources in the case study are presented below.

### 3.1 GPT-4’s ability to respond to a multiple-choice exam in medicine (MD4062)

The scoring of the responses to the 110 exam questions is based on the answer key/grading guidelines (“sensorveiledning”) for MD4062 in medicine (Norges teknisk-naturvitenskapelige universitet (22). Interaction with GPT-4 was conducted based on the 110 exam questions that were posed to GPT-4, and the responses were recorded (each response was considered final) (see Figure 1).

**Figure 2:**
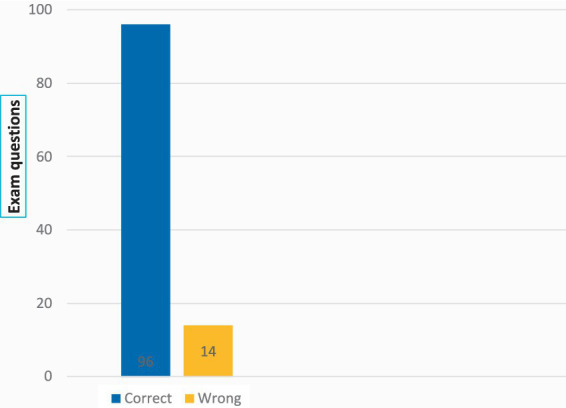
GPT-4’s responses to 110 exam questions. Accuracy Rate: GPT-4 answered 96 out of 110 questions correctly, which yielded an accuracy rate of approximately 87.27% (96/110 × 100). Error Rate: GPT-4 gave 14 incorrect answers, which resulted in an error rate of approximately 12.73% (14/110 × 100).

An accuracy rate of 87.27% indicates a strong capability/understanding of the material covered in the exam. The high number of correct answers reflects insights into and knowledge of the topic, and it is interesting that GPT-4 is capable of respond well to Norwegian exam questions (only a few weeks after it was launched in March 2023). GPT-4 not only master to give correct answers to the majority of the exam questions, but also explain why these answers are correct (with explanations of between 200 to 400 words for each question). These results reveal that GPT-4 would have passed this exam if it were an authentic exam context. While the performance is strong, we should bear in mind that this is a multiple-choice exam, and the 14 incorrect answers suggest there are still areas that need improvement (e.g., semantic misinterpretations of Norwegian expressions). However, the good results are in line with the current state of knowledge internationally (Figure 2).

### 3.2 GPT-4’s ability to respond to a multiple-choice exam (blinded alternatives)

The scoring of the responses to the 10 exam questions with blinded alternatives is based on the answer key/grading guidelines (“sensorveiledning”) for MD4062 in medicine. Interaction with GPT-4: 10 questions were posed to GPT-4 based on chain-of-thought prompting, and responses were recorded (each response was considered final; see Table 1).

**Table 1:** Distribution of right or wrong answers on multiple choice questions with blinded alternatives.

Exam-questions	*N*	Performance	Percentage of exam-assignments
Number 13	1	100	0.00909
Number 24	1	100	0.00909
Number 36	1	100	0.00909
Number 43	1	0	0
Number 52	1	100	0.00909
Number 61	1	0	0
Number 76	1	100	0.00909
Number 89	1	0	0
Number 95	1	100	0.00909
Number 105	1	100	0.00909

Example of the exam question (originally in Norwegian, MD4062):

*A 41-year-old woman consults you with acute onset facial palsy on the left side. She has a drooping left corner of her mouth and is unable to close her left eye. An MRI of the head (T1, T2, diffusion-weighted images) shows a large macroadenoma growing into the cavernous sinus on the left side, otherwise normal findings. She wonders if there could be a connection between the pituitary tumor and the facial palsy. What is the correct response to the patient?*

GPT-4 answered 7 out of 10 questions correctly, with an accuracy rate: 70% (7/10 × 100) and an error rate of 30% (3/10 × 100). An accuracy rate of 7 out of 10 on blinded alternatives on this exam indicates a solid understanding of the material. Most of the answers are correct, thereby demonstrating GPT-4’s overall competence in the subject matter. Additionally, GPT-4 not only excels at providing correct answers to the majority of exam questions (even with blinded exam options) but also explains why these answers are correct. However, a clear limitation here is that only 10 such blinded exam questions were tested, which is a significant weakness of this portion of the study.

### 3.3 GPT-4’s ability to analyze test results from a National Health Laboratory

Scoring of the responses to the two cases is based on the validity of community responses and quality assurance. Interaction with GPT-4: two cases were posed to GPT-4 based on chain-of-thought prompting, and responses were recorded (each response was considered final; see Table 1).

*Case 1*. Chain-of-thought prompting: *“Assess the health of this person based on the following information below. Provide detailed comments on areas where the person can improve their overall health, physical activity, diet, and sleep. Offer specific suggestions on how the person can improve their health with examples from the information provided. Age 42 years, resting pulse 48, blood pressure 120/80, does not smoke, no alcohol, sleeps 7–8 h per night, walks/runs 2000 kilometers per year, B-hemoglobin 15.3 g/dL, B-leukocytes 5.1 10^9/L, S-creatinine 62 umol/L, S-eGFR (CKD-EPI) 105, S-ALAT 34, B-HbA1c 38, S-triglycerides non-fasting 0.98, S-cholesterol 6.1, S-HDL-cholesterol 1.4, S-LDL-cholesterol 5.2, S-PSA 0.81.”*

GPT-4 assessed the abovementioned health data and provided the following response:

“Based on the provided information, this 42-year-old person appears to have a generally healthy lifestyle and good health. However, there are a few areas that can be improved. Here is a detailed evaluation:

1. *Resting pulse*: A resting pulse of 48 is slightly low for the average person, but can be normal for those who are physically active and in good shape. If this person experiences symptoms such as dizziness, unusual fatigue, or frequent headaches, they should discuss this with a health care professional.
2. *Blood pressure*: A blood pressure of 120/80 is within the normal range, which is a sign of good heart health.
3. *Smoking and alcohol*: This person does not smoke or drink alcohol, which is positive for their health.
4. *Sleep*: The person gets 7–8 h of sleep each night, which is within the recommended range for adults. This contributes to good sleep hygiene and overall health.
5. *Physical activity*: This person walks or runs approximately 2000 kilometers per year, which averages about 5.5 kilometers per day. This is a good amount of physical activity, but there is always room to increase it if possible.
6. *Blood test results*: All blood values—including B-hemoglobin, B-leukocytes, S-creatinine, S-eGFR, S-ALAT, B-HbA1c, and S-PSA—are within normal ranges. This indicates good kidney and liver function, good blood sugar regulation, and low risk of prostate cancer.
7. *Cholesterol and triglycerides*: Although HDL cholesterol and triglycerides are within normal ranges, total cholesterol, particularly LDL cholesterol, are slightly high. This can increase the risk of heart disease over time.

Here are some suggestions for improvements:

- *Cholesterol*: To lower LDL cholesterol and total cholesterol, the person should consider dietary changes. They can reduce the intake of saturated and trans fats, increase the intake of fiber-rich foods, and add more fish (especially fatty fish like salmon and mackerel) to their diet. Increased physical activity can also help improve cholesterol levels.
- *Medical follow-up*: Even though the health indicators look good, it is important to have regular medical check-ups to monitor blood pressure, blood sugar, and cholesterol levels.

Remember that it is always important to discuss health issues and lifestyle changes with a health care professional for a more accurate evaluation and guidance” (23).

*Case 2*. Chain-of-thought prompting: *“Assess the health of this person based on the information given below. Provide detailed comments on areas where the person can improve their overall health, physical activity, diet, and sleep. Offer specific suggestions on how the person can improve their health with examples from the provided information. Age 42 years, resting pulse 68, blood pressure 160/100, smoker, 2–3 units of alcohol per day, sleeps 5–6 h per night, walks 1,000 kilometers per year, B-hemoglobin 15.3 g/dL, B-leukocytes 5.1 10^9/L, S-creatinine 62 umol/L, S-eGFR (CKD-EPI) 105, S-ALAT 34, B-HbA1c 38, S-triglycerides non-fasting 0.98, S-cholesterol 6.1, S-HDL-cholesterol 1.4, S-LDL-cholesterol 5.2, S-PSA 2.8.”*

GPT-4 assessed these details and provided the following response:

“Based on the provided information, this person has several health concerns that should be addressed. Here is a detailed evaluation:

1. *Resting pulse*: A resting pulse of 68 is within the normal range but is on the higher end.
2. *Blood pressure*: A blood pressure of 160/100 is high (hypertension). Persistent high blood pressure can lead to serious health issues, including heart disease and stroke.
3. *Smoking*: Smoking is a major risk factor for many serious health problems, including heart and lung disease, and various cancers.
4. *Alcohol*: Consuming two to 3 units of alcohol per day exceeds the recommended daily intake and can contribute to numerous health issues, including liver disease and addiction.
5. *Sleep*: This person gets only five to 6 h of sleep per night, which is less than the recommended seven to 9 h for adults. Insufficient sleep can have numerous negative health effects, including increased risk of heart disease, diabetes, and a weakened immune function.
6. *Physical activity*: This person walks approximately 1,000 kilometers per year, which averages to about 2.7 kilometers per day. This is below the recommended level for good health.
7. *Blood test results*: Most of these values—including B-hemoglobin, B-leukocytes, S-creatinine, S-eGFR, S-ALAT, and B-HbA1c—are within normal ranges, which is good news. However, there are concerns regarding cholesterol levels, particularly LDL (“bad”) cholesterol, which is slightly high.
8. *S-PSA*: A PSA value of 2.8 is within the normal range for a 42-year-old man, but it may be wise to monitor this regularly.

Here are some suggestions for improvements:

- *Quit smoking*: This is the most important step the person can take to improve their health. There are various methods and resources available to help with quitting smoking, including nicotine replacement products and counseling.
- *Reduce alcohol intake*: This person should try to reduce their alcohol consumption to recommended levels or lower. This will also help to reduce blood pressure.
- *Improve sleep*: The person should aim to get at least 7 h of sleep each night. Good sleep habits can include maintaining a consistent sleep schedule, avoiding screens before bedtime, and ensuring the bedroom is dark and quiet.
- *Increase physical activity*: While some physical activity is better than none, the person should try to increase this, ideally to at least 150 min of moderate-intensity activity per week, or 75 min of high-intensity activity.
- *Change diet*: Eating a healthy diet can help reduce cholesterol levels and blood pressure. This should include eating plenty of fruits and vegetables, whole grains, lean protein sources, and limiting salt and sugar.
- *Medical follow-up*: With high blood pressure and slightly elevated cholesterol levels, this person should discuss treatment options with a health care professional; this may include medication in addition to lifestyle changes.

Remember that it is important to consult with a healthcare professional before starting or changing any treatment or lifestyle modification” (23).

The validity community generally assessed this feedback from GPT-4 as reliable and trustworthy on a general level for students in an educational context. They believe that the assessments provided by GPT-4 for both cases demonstrate the AI’s ability to analyze comprehensive health data and offer detailed recommendations for improvement.

*Case 1*: The patient is generally healthy, with minor adjustments needed for cholesterol levels. Recommendations focus on dietary changes and maintaining regular check-ups.

*Case 2*: The patient has several significant health risks, including hypertension, smoking, high alcohol consumption, insufficient sleep, and low physical activity. Recommendations include lifestyle changes, dietary adjustments, and medical follow-up.

Implications for GPT-4 in formative and summative assessments (e.g., medical exams and clinical practice):

- GPT-4 shows potential in providing accurate health assessments and practical recommendations, which aligns with established medical guidelines.
- GPT-4’s ability to analyze complex health data and generate comprehensive responses highlights its usefulness as a support tool in medical education and clinical practice.

Overall, GPT-4’s performance in these two cases suggests that it could be a valuable asset in medical education and in education in general, as it offers detailed and personalized feedback to improve patient outcomes through case simulations.

### 3.4 Validation communities for the results

The scale below was used by the validation community to assess the degree to which they agree with the accuracy and relevance of GPT-4’s responses. Each member rated the responses on this scale, and the results were aggregated to provide a comprehensive evaluation of the model’s performance. Below is an explanation of the Likert scale for degree of confirmation:

Strongly disagree: The response does not align with the expected answer at all; there are significant inaccuracies or misconceptions.
Disagree: The response has a few correct elements, but overall, it does not satisfactorily align with the expected answer.
Neutral: The response is somewhat accurate but lacks sufficient detail or context to be fully satisfactory.
Agree: The response is mostly accurate and aligns well with the expected answer, with minor omissions or inaccuracies.
Strongly agree: The response completely aligns with the expected answer, demonstrating high accuracy and relevance.

The validation communities mostly agreed or strongly agreed with the case results and findings but had several other concerns regarding the use of GPT-4 in student learning contexts (particularly ethical issues, plagiarism, etc.). The dialog with GPT-4 as a validation of its own performance retrospectively in autumn 2023 confirmed the test results in general, but also found more in-depth reasons responsible for the capabilities of GPT-4 in handling this exam so well.

### 3.5 Fieldwork

Further fieldwork from August 2023 to September 2024 involved interactions with GPT-4 to check for possible biases and misinterpretations and was conducted in light of the abovementioned knowledge summary within this field. This process revealed that GPT-4 had improved in a few areas, likely due to ongoing fine-tuning by OpenAI.

Further, during this period, I conducted extensive evaluations to identify potential biases in GPT-4’s responses, particularly focusing on racial, gender, and socioeconomic biases that have been documented in previous versions of AI models. This involved comparing GPT-4’s responses to diverse medical case studies and real-world scenarios to ensure that its recommendations were equitable and free from discriminatory patterns.

Additionally, the fieldwork included testing GPT-4’s performance—based on chain-of-thought prompting in interpreting complex medical data, such as patient histories and diagnostic results—to ensure accurate and contextually relevant responses. This was crucial in assessing the reliability of GPT-4’s diagnostic suggestions and its ability to support clinical decision-making processes.

The improvements noted during this period suggest that GPT-4’s enhanced training data and fine-tuning positively impacted its performance. These enhancements are critical, in combination with chain-of-thought prompting, to ensure that AI tools like GPT-4 can be effectively integrated into medical education and practice, thereby providing reliable support for both students and professionals.

As an additional validation step, acknowledging that GPT-4 is becoming an outdated model, we conducted a comparative analysis of GPT-4 against GPT-4o on the same exam questions. This analysis revealed that GPT-4o performed 5.2% better (92,47% correct answers) than GPT-4, indicating an improvement in model capabilities. Future research could further validate results by comparing other advanced LLMs (e.g., Claude, LLAMA, Grog), enhancing the reliability and generalizability of findings in this rapidly evolving field.

Overall, while the limited scope of the case study remains a drawback, the continuous refinement and validation of GPT-4’s capabilities emphasize its potential to significantly enhance educational outcomes in the medical field.

## 4 Conclusion

The research questions in this case study focused on addressing GPT-4’s capabilities in terms of responding on a Norwegian medicine exam in the sixth year of medicine education in Norway (with Norwegian exam questions) a few weeks after it was launched (March 15, 2023). In the following section, I sum up the findings of the study and conclude based on the research questions of the study:

### 4.1 What is the capability of GPT-4 in answering a medicine exam in Norway?

The high accuracy rate of 87.27% (in Section 3.1) and an accuracy rate of 7 out of 10 (in Section 3.2) demonstrates GPT-4’s strong capability and understanding of the material covered in the medicine exam. This performance is very good, particularly given that GPT-4 responded effectively to Norwegian language exam questions only a few weeks after its launch in March 2023. The high number of correct answers reflects substantial insights and knowledge regarding the subject matter. It is also worth noting that GPT-4 not only excels at providing correct answers to the majority of exam questions (summative assessment) but also offers a form of formative assessment by explaining why these answers are correct and, if asked, why others are wrong. However, the study found that GPT-4 failed to correctly respond to 14 out of 110 questions, thereby resulting in an accuracy rate of 87.27%. Upon analysis, it was observed that these 14 failed responses did not follow a discernible pattern nor were they from a specific category. This suggests that the failures were distributed across various topics and types of questions. Several factors could contribute to these incorrect responses. It may be that a few questions required a higher level of nuanced understanding or context that GPT-4 could not fully grasp. Further, questions with ambiguous wording, specific Norwegian expressions, or multiple possible interpretations might lead to incorrect answers. The model’s training data may not sufficiently cover certain specific scenarios or medical cases, thereby leading to gaps in its knowledge. Despite these failures, the high overall accuracy indicates strong performance. Future research should further investigate these aspects to identify specific improvements. Understanding the reasons underlying these failures is crucial for enhancing the model’s reliability and accuracy in medical assessments.

Furthermore, an accuracy rate of 7 out of 10 on blinded alternatives on the medicine exam suggests a solid understanding of the material. The use of blinded alternatives helps ensure that the results accurately reflect GPT-4’s actual understanding without biases. This method can be useful in assessing knowledge and identifying areas that need improvement. However, the limitation of only 10 blinded questions in this study implies that the results may not fully capture the model’s performance variability. Future analysis should focus on identifying specific questions that are incorrectly answered to determine if there are patterns or common topics in which GPT-4 tends to make mistakes. Overall, GPT-4 shows strong potential in handling a medicine exam (summative assessment) in Norwegian; however, further refinement and analysis are needed to optimize its performance and reliability in educational settings.

Additionally, an important next step for validation would be to increase the number of blinded questions substantially and employ multiple sets of independently curated questions. Performing validation with larger datasets, perhaps involving various subject matter experts, would enhance the robustness and generalizability of the findings. Comparative studies involving human examiners or other AI models could also provide further insights into GPT-4’s relative strengths and weaknesses. Overall, GPT-4 shows strong potential in handling a medicine exam (summative assessment) in Norwegian; however, further refinement and analysis are needed to optimize its performance and reliability in educational settings.

### 4.2 How can GPT-4’s capability be applied to deal with formative and summative assessments for students?

As we have seen in this study, summative assessments evaluate cumulative knowledge at the end of a learning period, typically through exams or final projects, with the goal of measuring overall achievement. In contrast, formative assessments provide ongoing feedback throughout the learning process, aiming to improve student learning and guide future performance. In this study, GPT-4 addresses both types of assessments in distinct ways: for formative assessments, it offers adaptive, real-time feedback to help students improve as they learn; for summative assessments, it functions as a tool for exam preparation by providing interactive feedback based on previous exam sets, thereby aiding in evaluating cumulative knowledge. Then, what are the key findings of the study?

GPT-4’s performance in the two cases (in Section 3.3.) presented in this paper suggests that it could be a valuable asset in medical education and in education in general as a sparring partner, offering detailed and personalized feedback to improve patient outcomes through case simulations. In light of the high accuracy rate of 87.27% (in Section 3.1) and a rate of 7 out of 10 (in Section 3.2), Section 3.3 demonstrates GPT-4’s strong capability and understanding of the material covered in the medicine exam and in patient cases. Together, Sections 3.1, 3.2, and 3.3 highlight new opportunities for formative assessment, thereby showcasing GPT-4’s ability to provide accurate feedback, effectively handle blinded questions, and assess complex, authentic cases. As we have seen GPT-4 not only excels at providing correct answers but also by explaining why these answers are correct and why others are wrong. This is crucial for students because GPT-4 not only provides correct answers but also deepens their understanding by offering explanations for why those answers are correct. This interactive feedback helps students grasp underlying concepts, reinforcing learning rather than simply memorizing facts. Moreover, when students inquire why other options are wrong, they gain a clearer understanding of common misconceptions, sharpening their critical thinking and problem-solving skills. This form of formative assessment fosters a more thorough comprehension of the subject, promoting active learning and self-improvement, but also require a sufficient chain of thought prompting by the students. This comprehensive approach supports the potential for enhancing formative assessment practices in educational settings. This is important in students’ learning processes and is in line with the current state of knowledge in the context of formative and summative assessments (17, 18). GPT-4 can be used to provide students with personalized evaluations of their performance. Since GPT-4 can analyze individual answers and provide insights into why certain responses were incorrect, this is in line with ITS and one-on-one tutoring (15). VanLehn highlights that human one-to-one tutoring has long been considered one of the most effective forms of instruction, often referred to as the “gold standard.” He compares the efficacy of ITS to human tutors, noting already in 2011 that while ITS do not yet match the effectiveness of expert human tutors, they can provide comparable learning gains in many cases, particularly when designed to simulate one-to-one tutoring interactions. One of the key findings in this study is that GPT-4 can effectively scaffold learning and assist in developing customized study plans that focus on areas in which individual students need improvement. This aligns with the principles of ITS (15, 16), which emphasize formative assessments to optimize learning. By identifying specific gaps in knowledge and tailoring feedback to those needs, GPT-4 helps to make studying more efficient and effective, thereby enhancing student outcomes.

As we have seen in Sections 3.1 and 3.2, GPT-4 is capable of functioning as a sparring partner when students are preparing for summative assessments (e.g., exams) and create a new coherence between formative assessment practices and summative assessment practices. The theoretical underpinning of the study emphasizes the importance of such alignment among different assessment practices (17).

By analyzing the results of summative assessments, GPT-4 can provide comprehensive performance analytics, identifying trends and patterns in student performance across different cohorts. This data can be used to improve teaching strategies and curriculum design. Summative assessments can highlight areas where students consistently struggle, thus enabling educators to adjust their teaching methods or provide additional support to address these gaps. However, both the current state of knowledge and the validity community concerns regarding aspects such as plagiarism and AI as an “ethical minefield” is also an obvious aspect of this discourse and require a high vigilance of such problematic issues in educational and assessment practices.

One of the interesting findings of the study was GPT-4’s ability to handle the Norwegian language during the six-month testing period from March 25, 2023, to August 8, 2023. The AI’s strong grasp of the Norwegian language not only facilitated accurate summative assessment but also enabled the provision of detailed, personalized feedback. This proficiency emphasizes the potential of GPT-4 as an intelligent AI-tutor and versatile tool in diverse linguistic and educational contexts, offering significant support for both students and educators in Norway and Norwegian-speaking regions worldwide. These findings align with the results from a Norwegian study by Mork et al. (24), who similarly found that GPT-4 ability to assist healthcare professionals by generating high-quality initial responses to health-related questions in Norwegian.

Overall, this study aimed to explore GPT-4’s capabilities in a medical educational setting, particularly in terms of formative and summative assessments. The findings demonstrate that GPT-4 is a promising tool for enhancing assessment practices, as evidenced by its performance on a full-scale medical multiple-choice exam in Norwegian educational settings. The results align with previous research by Gilson et al. (10), Nori (9) and Kung et al. (11), which found that large language models, including GPT-4, can perform at or near the passing threshold for medical exams such as the NBME-Free-Step-1 and the United States Medical Licensing Exam (USMLE). Like these studies, our research demonstrates that GPT-4 performed well in summative assessments, showing a robust ability to handle the nuances of the Norwegian medical language and providing reliable evaluations of complex patient cases.

A significant contribution of this study lies in its exploration of GPT-4’s potential for formative assessments. The model’s capacity to provide timely, personalized feedback to students positions it as a valuable tool for supporting student learning. Madrid et al. (14) similarly noted that GPT-4, when supplemented with specialized plugins, was able to offer logical justifications and personalized feedback, enhancing its application in educational contexts. This suggests that GPT-4 not only serves as a reliable summative assessment tool but also plays a crucial role in formative assessment, which is critical for ongoing student development.

Moreover, the study supports findings by Grévisse (13), who demonstrated that GPT-4’s precision in grading correct answers closely matched human evaluations. However, Grévisse found that GPT-4 tended to assign lower scores compared to human evaluators, a trend not fully observed in our study. In our research, GPT-4’s performance on summative assessments, including comprehensive evaluations of patient cases, aligned closely with human assessments (the validity community), suggesting that the model is capable of handling complex medical scenarios. This reinforces the argument that GPT-4 can be effectively integrated into medical education for tasks requiring nuanced judgment.

In addition to the empirical findings, this study contributes to the theoretical discourse on the role of AI in education, particularly regarding the importance of chain-of-thought prompting and scaffolding in tutoring and assessment practices (17). Gilson et al. (10) emphasized the dialogic nature of GPT-4’s feedback, which provides students with not only answers but also reasoning and informational context, a capability that enhances formative learning. The ability of GPT-4 to scaffold student learning by offering structured, logical reasoning in its feedback strengthens its potential as an Intelligent Tutoring System (ITS) (15, 16) in medical education.

However, it is important to acknowledge the limitations of GPT-4 identified in this and other studies, particularly the risk of perpetuating biases and ethical concerns. As noted by Artsi et al. (12), while GPT-4 and other LLMs show promise in generating valid medical exam questions, they are not without their limitations, and there is a need for ongoing refinement to ensure accuracy and fairness. In our study, similar limitations were also evident, emphasizing the importance of careful implementation and continuous oversight to mitigate potential biases in both formative and summative assessments.

In conclusion, this study contributes to the growing body of literature on the application of large language models like GPT-4 in medical education (25, 29–32). While GPT-4 shows substantial potential in enhancing both formative and summative assessments, further research is needed to address the ethical and practical challenges associated with its use. Continuous refinement and human oversight remain crucial to ensure the effective and responsible integration of AI technologies into educational settings.

### 4.3 Limitations

This study used GPT-4 with a default temperature setting of 0.7, allowing for a certain amount of variability in responses. Only a single iteration per question was performed, and the results were accepted as final. Future studies could use a temperature setting of 1.0 and multiple iterations to better evaluate consistency and provide a more robust statistical analysis of GPT-4’s accuracy in summative assessments.

An important consideration in the use of LLMs such as GPT-4 in medical and educational settings is the “black box” problem. This issue refers to the opacity in how these models make decisions or generate responses, thus making it difficult to understand the underlying reasoning processes. This lack of transparency can be problematic, particularly in high-stakes environments—such as education and health care—where reliability and accountability are paramount. The black box problem poses significant challenges, including difficulties in debugging and trust-building among users. In educational settings, students and educators may find it difficult to trust or learn from AI-generated feedback if the reasoning underlying the responses is unclear. Similarly, in medical contexts, health care professionals need to understand the basis of AI-generated diagnoses or treatment recommendations to ensure patient safety and provide informed care.

Approaches such as explainable AI (XAI) and uncertainty quantification offer promising solutions to these challenges. Explainable AI aims to make AI models more transparent by providing insights into how decisions are made, thereby enhancing trust and facilitating better decision-making. For example, incorporating explainable AI techniques can help educators and health care professionals understand the rationale underlying GPT-4’s responses, thereby making it easier to verify and rely on the information provided (14).

Uncertainty quantification is another approach that can help mitigate the limitations of black box models. By quantifying the uncertainty associated with AI predictions, this method enables users to gage the confidence level of the responses generated by the model. This is particularly useful in medical settings, in which understanding the uncertainty of a diagnosis or treatment recommendation can guide further investigations and decision-making processes (14). Incorporating these approaches can significantly enhance the usability and reliability of AI systems such as GPT-4 in medical and educational settings. By addressing the black box problem through explainable AI and uncertainty quantification, we can improve transparency, build trust, and ensure that AI technologies are used safely and effectively in these critical domains.

Another critical consideration is the ethical challenges posed by AI. When implementing AI technologies such as GPT-4 in educational settings, it is essential to ensure compliance with regulations such as the General Data Protection Regulation (GDPR) (26) and the Artificial Intelligence Act (27). Norwegian regulations emphasize data protection, privacy, and the ethical use of AI, which is in line with these regulations. Madrid et al. (14) emphasize Europe’s stringent AI regulations, which focus on ensuring transparency, accountability, and the elimination of bias in AI systems. These regulations aim to protect users while fostering trust and innovation in AI technologies. The Norwegian framework similarly upholds these principles, which are vital for the ethical and feasible implementation of AI in education. Adhering to these standards enables the responsible integration of GPT-4 and AI in educational settings, thus promoting innovation while safeguarding the rights and interests of all stakeholders.

## Data Availability

The raw data supporting the conclusions of this article will be made available by the authors, without undue reservation.

## Footnotes

^1^ https://www.furst.no/
