# Supplementary material for: GPT-4’s capabilities for formative and summative assessments in Norwegian medicine exams—an intrinsic case study in the early phase of intervention
Source: Front Med (Lausanne). 2025 Apr 10;12:1441747. doi: 10.3389/fmed.2025.1441747 (PMC12018347; doi:10.3389/fmed.2025.1441747)
Supplement: Supplementary file 1 [file Data_Sheet_1.pdf]

Appendix 1. General chain-of-thought-prompting of the study

You are taking a comprehensive and final Medicine exam designed specifically for medical students, consisting of 110 multiple-choice questions. Each question is presented as a clinical patient case, challenging you to apply your medical knowledge in real-world scenarios.

For each clinical case-based multiple-choice question, adopt a methodical and comprehensive approach:

Patient Case Analysis:

- *Carefully read the clinical scenario presented. Identify key information, including the patient's symptoms, medical history, examination findings, and any diagnostic test results.*
- *Relate the case details to the underlying pathophysiology and identify the most probable diagnosis or clinical issue.*

- **Critical Evaluation of Each Option:**
  - *Systematically assess each answer choice. For the correct answer, explain why it is the most appropriate based on the clinical presentation, referencing relevant anatomical, physiological, pathological, or pharmacological principles.*
  - *For the incorrect options, discuss why they do not fit the clinical picture. If relevant, explain common pitfalls or how these options might be applicable in different clinical contexts.*
- **Application of Medical Knowledge:**
  - *Integrate the biological, anatomical, and physiological principles into the context of the patient's case. Consider how the clinical findings correspond to the pathophysiology of the suspected condition and how each answer choice could influence patient management.*
- **Connection to Real-World Clinical Practice:**
  - *Reflect on how the correct understanding of the case and the underlying medical principles would guide clinical decision-making. Consider the steps you would take as a physician to diagnose, manage, or treat the patient based on the correct answer.*
  - *Explore how this knowledge applies in patient care and how recognizing the correct diagnosis or treatment option could improve patient outcomes.*
- **Rationale for Enhancing Patient Care:**
  - *Consider how this clinical knowledge and its application will enhance your ability to provide effective, patient-centered care. Discuss the potential clinical implications of misdiagnosing or mismanaging a similar patient case, and how mastery of these concepts ensures safe and optimal treatment.*
- **Comprehensive Reasoning:**
  - *Demonstrate a well-rounded approach to clinical reasoning by explaining your thought process. Show not only why the correct answer fits the case, but also why the other options are less likely or inappropriate given the patient's presentation.*

- *Avoid focusing solely on identifying the correct answer. Instead, emphasize the clinical reasoning process, patient-centered care, and the impact of the medical decisions being assessed.*

- This exam evaluates not just your ability to recall facts, but also your capacity to synthesize clinical information, apply medical knowledge to real-world scenarios, and make sound decisions that improve patient outcomes.
